# Rapid Assessment of Malaria Transmission Using Age-Specific Sero-Conversion Rates

**DOI:** 10.1371/journal.pone.0006083

**Published:** 2009-06-29

**Authors:** Laveta Stewart, Roly Gosling, Jamie Griffin, Samwel Gesase, Joseph Campo, Ramadan Hashim, Paul Masika, Jacklin Mosha, Teun Bousema, Seif Shekalaghe, Jackie Cook, Patrick Corran, Azra Ghani, Eleanor M. Riley, Chris Drakeley

**Affiliations:** 1 Department of Infectious and Tropical Diseases, London School of Hygiene and Tropical Medicine, London, United Kingdom; 2 Joint Malaria Programme, Moshi, Tanzania; 3 KILI-IPTi Project, JMP, Korogwe, Tanzania; 4 MRC Centre for Outbreak Analysis and Modelling, Department of Infectious Disease Epidemiology, Imperial College, London, United Kingdom; 5 Kilimanjaro Christian Medical College, Tumaini University, Moshi, Tanzania; 6 Department of Medical Microbiology, Radboud University Nijmegen Medical Centre, Nijmegen, The Netherlands; 7 Biotherapeutics Group, NIBSC, South Mimms, Herts, United Kingdom; Walter and Eliza Hall Institute of Medical Research, Australia

## Abstract

**Background:**

Malaria transmission intensity is a crucial determinant of malarial disease burden and its measurement can help to define health priorities. Rapid, local estimates of transmission are required to focus resources better but current entomological and parasitological methods for estimating transmission intensity are limited in this respect. An alternative is determination of antimalarial antibody age-specific sero-prevalence to estimate sero-conversion rates (SCR), which have been shown to correlate with transmission intensity. This study evaluated SCR generated from samples collected from health facility attendees as a tool for a rapid assessment of malaria transmission intensity.

**Methodology and Principal Findings:**

The study was conducted in north east Tanzania. Antibodies to *Plasmodium falciparum* merozoite antigens MSP-1_19_ and AMA-1 were measured by indirect ELISA. Age-specific antibody prevalence was analysed using a catalytic conversion model based on maximum likelihood to generate SCR. A pilot study, conducted near Moshi, found SCRs for AMA-1 were highly comparable between samples collected from individuals in a conventional cross-sectional survey and those collected from attendees at a local health facility. For the main study, 3885 individuals attending village health facilities in Korogwe and Same districts were recruited. Both malaria parasite prevalence and sero-positivity were higher in Korogwe than in Same. MSP-1_19_ and AMA-1 SCR rates for Korogwe villages ranged from 0.03 to 0.06 and 0.07 to 0.21 respectively. In Same district there was evidence of a recent reduction in transmission, with SCR among those born since 1998 [MSP-1_19_ 0.002 to 0.008 and AMA-1 0.005 to 0.014 ] being 5 to 10 fold lower than among individuals born prior to 1998 [MSP-1_19_ 0.02 to 0.04 and AMA-1 0.04 to 0.13]. Current health facility specific estimates of SCR showed good correlations with malaria incidence rates in infants in a contemporaneous clinical trial (MSP-1_19_ r^2^ = 0.78, p<0.01 & AMA-1 r^2^ = 0.91, p<0.001).

**Conclusions:**

SCRs generated from age-specific anti-malarial antibody prevalence data collected via health facility surveys were robust and credible. Analysis of SCR allowed detection of a recent drop in malaria transmission in line with recent data from other areas in the region. This health facility-based approach represents a potential tool for rapid assessment of recent trends in malaria transmission intensity, generating valuable data for local and national malaria control programs to target, monitor and evaluate their control strategies.

## Introduction

Recent years have seen the widespread implementation of various malaria control strategies including the use of insecticide impregnated mosquito nets (ITN), insecticide residual spraying (IRS) and artemisinin combination therapies (ACT) [1]–[3]. Moreover, there is evidence that malaria transmission is decreasing in several areas in sub-Saharan Africa [4]–[8]. These successes, together with a considerable increase in funds available for malaria control activities, have sparked renewed optimism for malaria elimination programmes [9], [10]. A key element in focusing control and elimination efforts will be obtaining reliable measures of malaria transmission intensity (MTI) which is a crucial determinant of the burden of malaria disease [11]–[13]. However, it is not clear how best to monitor changes in transmission and disease burden [14]. Moreover, transmission of malaria is notably heterogeneous and different control measures may be better suited to different transmission intensities. Similarly, different methods (and combinations of methods) with differing provenance and characteristics will be needed for measuring transmission at different levels [12]. As such, a technique that generates locally applicable measures of MTI and in a rapid, logistically feasible manner would have great potential for use by district and national malaria control teams.

The current gold-standard for measuring MTI is the Entomological Inoculation Rate (EIR), determined as the number of infectious bites per person per year (ib/p/yr). EIRs vary across Africa, ranging from less than one to greater than 1000 ib/p/yr [15]. Despite its undoubted relevance and provision of important information on mosquito species and temporal dynamics, determination of EIR is not suited to obtaining rapid estimates of MTI. It is typically a laborious and time-consuming method; moreover mosquito distributions are notably heterogeneous [16]–[18] with intensive sampling required to provide robust estimates at fine scale and at low mosquito densities. Parasite prevalence (PR), estimated by microscopy or, increasingly, by Rapid Diagnostic Tests (RDTs), has similar limitations. Whilst PR can be estimated reasonably quickly, the resulting prevalence is limited by the sensitivity of the assay used and estimates can be profoundly influenced by anti-malarial drug intake and the timing of collection, especially in areas of seasonal transmission; PR also has limited sensitivity to measure changes at the high and low ends of the spectrum of transmission intensities [19]. Estimation of PR using molecular techniques has increased sensitivity but is time consuming and requires skills and equipment unlikely to be available in many resource-poor settings.

An alternative measure of MTI is the prevalence of anti-malarial antibodies in the local population. Previously, a sero-epidemiological approach to evaluate exposure to malaria was widely advocated but its uptake was limited by the requirement for cultured parasites as a source of antigen and a lack of objective criteria for determining sero-positivity [20]–[22]. More recently, objective analysis of ELISA data (obtained using recombinant malaria antigens) has shown considerable promise, with antibody sero-conversion rates (SCR) showing tight correlation with EIR [23]. The advantages of this approach are several. Antibody responses represent an integration of malaria exposure over time and allow an evaluation of temporal trends in transmission. The longevity of the antibody response generates sero-prevalences that are higher than equivalent parasite rates and the method thus has greater sensitivity in low transmission settings. Technically, SCR assays are simple, cheap and quick to perform and can be adapted to different transmission settings by using antigens of differing immunogenicity [24]. One operational benefit is that antibodies can be eluted from filter paper thereby simplifying sample collection and storage in field conditions [25].

In this study we have examined the utility of serological markers of malaria exposure to rapidly assess MTI in an area of northeast Tanzania. A pilot study was conducted to compare sero-prevalence data from samples collected in cross-sectional surveys with samples from attendees at the health facility in the same community. The latter approach relies on sampling all individuals attending a health facility over a fixed period of time (or until the required number of samples have been obtained), including malaria and non-malaria cases and healthy companions. Sero-prevalence data collected across all age groups, within the context of a large scale intermittent presumptive treatment trial in infants (the KILI-IPTi study) [26], were converted into SCR and used to estimate current and recent trends in malaria transmission. The study demonstrated the utility of rapid assessment of SCR for obtaining realistic estimates of MTI and provided important contextual information to aid interpretation of the results from the IPTi study.

## Methods

### Study Site

The study was conducted in north east Tanzania where malaria transmission is associated with the two annual rainy seasons; there is marked small scale heterogeneity in malaria transmission associated with altitude and rainfall [27].

### Pilot study

**Figure 1:**
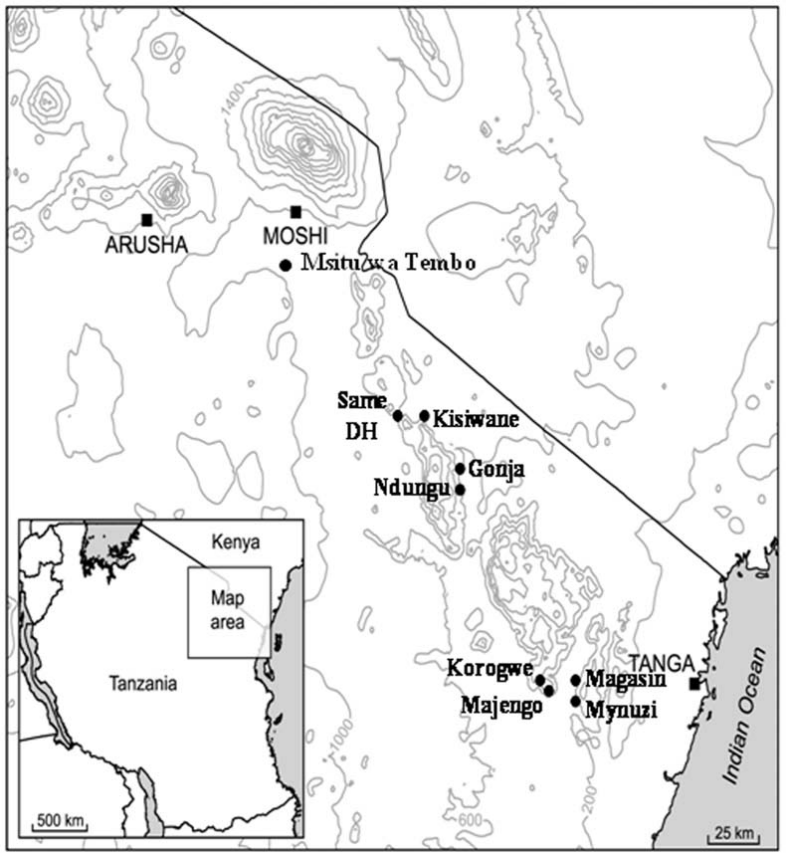
Map showing the study area: Squares mark major towns, circles mark villages with study dispensaries.

Samples were collected from subjects recruited at the Msitu wa Tembo health facility in lower Moshi (Figure 1) in a 3 week period in February 2007. This is a low malaria transmission area on the slopes of mount Kilimanjaro with an EIR of ∼3 ib/p/yr [28] where we have previously conducted a cross-sectional malaria prevalence surveys. Eligible participants were any attendee at the health facility for any reason (i.e. family members or guardians accompanying patients as well as patients themselves) who consented to take part. There were no age or gender restrictions. Study details were explained to all potential adult and guardians of child participants. Subsequently, signed or fingerprinted informed consent was obtained for adults and guardians of child participants prior to enrolment. A copy of the consent form was offered to all subjects. Staff at the health facility were informed of the study during a group sensitization session at both the Outpatient Department (OPD) and Maternal and Child Health (MCH) sections of the clinic. Study information sheets in Kiswahili were posted at the clinic.

Consenting participants were registered and assigned a unique ID number which corresponded to the district, dispensary and patient number. Additional information on age, sex and village of residence was collected. Blood samples were collected from finger pricks into an EDTA-coated micro-tube, onto filter paper (3MM Whatman) and onto a rapid diagnostic test (RDT; Parahit-f, Span Diagnostics Limited). RDT results were given to the prescribing officer to assist in patient management. Filter papers stored, after rigorous air drying, in a sealed container containing silica gel as a desiccant, as described previously [25] and were returned, with the EDTA blood sample, to the laboratory at Kilimanjaro Christian Medical Centre (KCMC) for same-day processing, storage and subsequent analysis.

Plasma samples were also obtained from a cross-sectional survey carried out in the village of Msitu wa Tembo in 2005 (for survey methodology see [29]).

### IPTi study

Subjects were recruited from health facilities within the Korogwe and Same Districts of Tanzania between July and August 2007. Korogwe district is a moderate transmission site located in the Tanga region and Same district is categorized as a low transmission site located to the north and further inland than Korogwe [27] (Figure 1). Four health facilities per district (Korogwe: Magunga, Majengo, Mnyuzi and Magasin; and Same: Same District Hospital, Kisiwani, Gonja Moare and Ndungu Health Centre) were selected based on their involvement within the Kili-IPTi study [26] (Figure 1).

Consenting participants were enrolled as for the pilot study and finger prick blood samples collected onto filter paper and RDTs. Filter paper samples were collected, dried, stored at 4°C in air tight containers with desiccant and transported to the KCMC biotechnology laboratory after ∼2 weeks.

### Samples for ELISA assays

#### I. Plasma

EDTA blood samples were centrifuged for 5 minutes (13,000 rpm) and the plasma removed and stored at −20°C. Prior to analysis plasma samples were diluted 1∶1000 with PBS containing 0.1%Tween 20 (Sigma, US).

#### II. Reconstitution of Filter Paper Blood Spots

Briefly, using a hole punch, a 2.5 mm blood spot (equivalent to approximately 1.5 µl of blood) was removed from the filter paper and placed into an appropriately labelled well of a low-binding 96 well titre plate. To each well, 150 µl of reconstitution buffer (PBS plus 0.05%Tween and 0.1% (w/v) sodium azide) was added. Sample plates were sealed and rocked gently at room temperature overnight before storage at 4°C until use. The reconstituted blood spot solution was equivalent to a 1/100 dilution of whole blood or 1/200 of serum (assuming a haematocrit of approximately 50%). Ultimately, the final serum dilution equivalent on the processed ELISA plate was 1/1000.

### Enzyme-linked immunosorbent assay

All test samples (plasma, reconstituted filter paper spots) were tested for anti-MSP-1_19_ and anti-AMA-1 human IgG antibodies by ELISA using standard methodology [23], [25]. Briefly, recombinant MSP-1_19_ (Wellcome genotype) and AMA-1 (3D7) were coated overnight at 4°C at a concentration of 0.5 µg/ml respectively. Plates were washed using PBS plus 0.05% Tween 20 (PBS/T) and blocked with 1% (w/v) skimmed milk powder in PBS/T. Samples, a positive (a pool of hyperimmune serum collected from a malaria endemic area) and negative controls (serum from European malaria naïve volunteers) were added in duplicate to each plate. After washing, horseradish peroxidase-conjugated rabbit anti-human IgG (DAKO) (1/5000 in PBS/T) was added to all wells. All plates were developed using OPD substrate solution and reactions were stopped with 2 M H_2_SO4. Plates were read immediately at 492 nm and optical density (OD) values recorded.

### Sample size calculations

Sample sizes were calculated by fitting an empirical function to the results of previous studies [23] to determine how the % relative standard deviation (%RSD; equivalent to the coefficient of variance) scales with the number of positive individuals sampled. A logarithmic function was fitted and used to calculate a sample size (N) necessary to generate sufficient sero-positive samples at different values of SCR to give the required %RSD. It was assumed that the low transmission site in Moshi and Same had a SCR of 0.05/yr (EIR∼3) and for a 20% RSD 237 samples are required. The equivalent value for the high transmission sites with an assumed SCR of 1.0 and 20% RSD was 160 samples.

These samples sizes were incorporated into a logistically viable operational approach which allowed for the varying levels of attendance at the different health facilities and for the fact that samples were collected over a similar time period. Ultimately a study pack containing sufficient data forms, filter papers, RDT's and storage boxes for 500 individuals was left at each health facility. Completed study packs and samples were collected from dispensaries once a week, during routine supervision visits.

### Statistical Methods

For each sample duplicate OD results were averaged and normalized against the positive control run on each plate. Antibody titre was estimated using the formula: titre = dilution/[maximum OD/(OD test serum- minimum OD)-1]. A cut off above which samples were deemed antibody positive was defined using a mixture model as previously described [25]. Briefly, the distribution of normalized OD values was fitted as the sum of two Gaussian distributions (a narrow distribution of sero-negatives and a broader distribution of sero-positives) using maximum likelihood methods. The mean OD of the Gaussian corresponding to the sero-negative population plus three standard deviations was used as the cut-off for sero-positivity (Cook et al, unpublished). A separate cut off was generated for each antigen (MSP-1_19_ & AMA-1), each sample type (plasma & filter paper) and each study (Pilot or IPTi). The sero-conversion rate (SCR or λ) was estimated by fitting a simple reversible catalytic model to the measured sero-prevalence, stratified into yearly age-groups, using maximum likelihood methods. For these models only individuals aged 1 and over were included to remove the effect of maternally-derived antibodies in infants. Additionally, evidence for temporal changes in SCR was explored by fitting models in which the SCR is allowed to change at a single time-point. The significance of the change was identified using likelihood ratio tests against models with no change, and profile likelihoods were plotted to determine confidence intervals for the estimated time of the change [30]. Lambda SCR values were converted to EIR equivalents using a log log regression equation based on previously collected SCR and EIR values from areas of different transmission intensity in Tanzania [23], [24]. Antigen-specific fixed values of rho, ρ, the sero-reversion rate, were fixed based on previous estimates from Tanzania [24](for MSP-1_19_ rho = 0.0173/yr & AMA-1 rho = 0.0262/yr). All analysis was carried out using Stata 10 (Statacorp, Texas US).

### Ethical approvals

Ethical approval for the study was obtained from the Tanzanian National Institute of Medical Research (NIMR/HQ/R.8a/Vol.IX./553), the Kilimanjaro Christian Medical Centre (KCMC: #224) and the London School of Hygiene & Tropical Medicine (LSHTM: #5136).

## Results

### Pilot study

**Figure 2:**
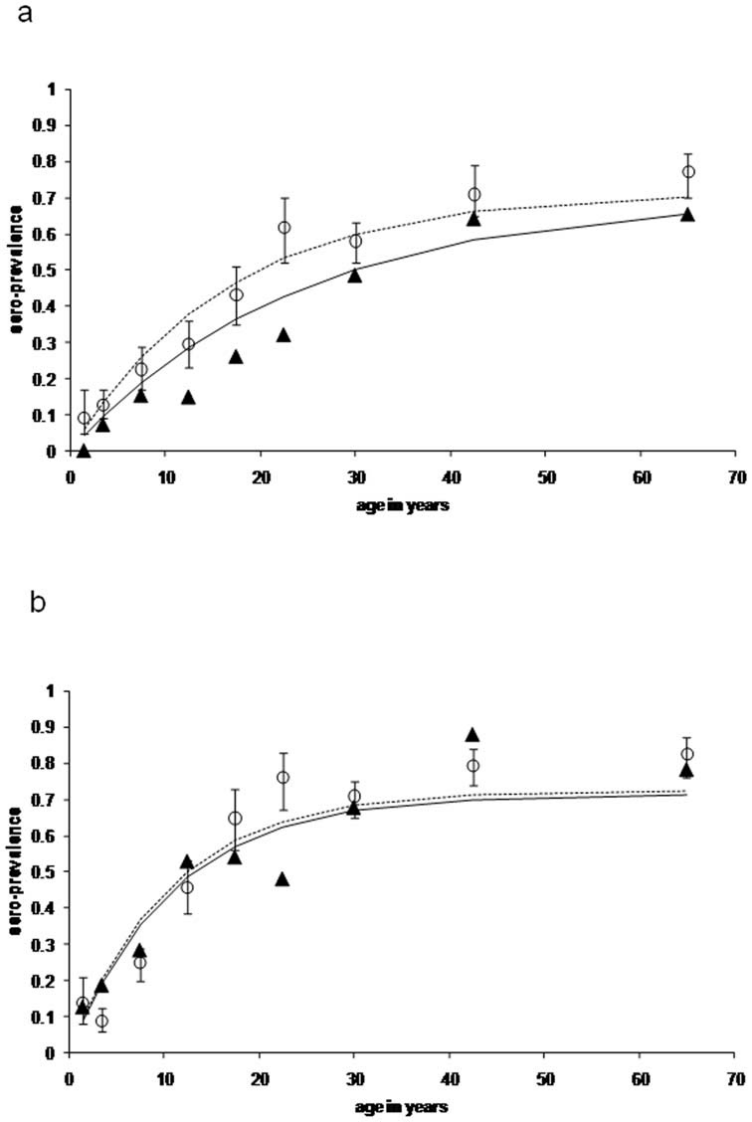
Age sero-prevalence plots for antibody responses to *P. falciparum* parasite antigens MSP-1_19_ (fig 2a) and AMA-1 (figure 2b) from the pilot study (Msitu wa Tembo). Open circles (and confidence limits) represent observed age group specific sero-prevalence points for the cross sectional survey. The dotted line represents a maximum likelihood fit using these data. The full triangles and unbroken line represent observed sero-prevalence points and fitted line for the health facility surveys.

For the pilot study, samples were collected from 341 individuals attending the health facility in Msitu wa Tembo over a 4 week period. Compared with the previous cross-sectional survey, the proportion of individuals under the age of 1 year was higher than expected (12.9% vs 4.5%, p<0.001) presumably as a result of infant attendance at the MCH clinic for routine vaccination (Table 1). Parasite positivity (as determined by RDT) was also higher in those recruited at the clinic compared with those recruited in the cross-sectional surveys (4.7% vs. 2.3%, p<0.001). Overall, sero-prevalence for MSP-1_19_ was significantly lower at the health facility than in the cross-sectional survey (29.1% vs. 40.5%, p = 0.005) but antibody sero-prevalence was similar for AMA-1 (46.9% vs. 47.9%, p = 0.8). No significant differences in anti-AMA-1 antibody titres were seen between subjects recruited at the health facility and and those recruited in the community. Among children aged under 1 year and children aged 2–5 years, mean MSP-1_19_ antibody titres were significantly higher among children recruited at the health facility than among children recruited in the community. However, in adults, anti-MSP-1_19_ titres were higher among those recruited in the community than among those recruited at the health facility (Table 1). In the community surveys, mean antibody titres were significantly higher in RDT positive than in RDT negative individuals for both antigens (MSP-1_19_: 216 vs 72 p<0.001; AMA-1: 161 vs 74 P<0.005). Antibody titres were also higher in RDT positives than RDT negatives in the health facility survey but not significantly so (MSP-1 titres 63 vs 49 p = 0.6; AMA-1 116 vs 65 P = 0.08). Age sero-prevalence plots for each antigen are shown in Figure 2. Estimated SCR values obtained from the statistical model, assuming no change in transmission intensity, for health facility and cross-sectional surveys were not significantly different for AMA-1 [ 0.63 (95% CI 0.52–0.76) and 0.61 (95% CI 0.56–0.65) respectively; p = 0.4] but the health facility estimate for MSP-1_19_ 0.24 (95% CI 0.19–0.29)] was significantly lower than the cross sectional estimate 0.42 (95% CI0.39–0.45; p = 0.012).

**Table 1:** Age distribution, RDT positivity and serological responses to both MSP-1_19_ and AMA-1 of patients recruited through cross-sectional and health facility surveys in Msitu wa Tembo.

Age group	Cross sectional survey			MSP-1_19_	AMA-1	Health facility survey			MSP-1_19_	AMA-1
	N (%)	RDT+				N (%)	RDT+			
<1	105 (4.4)	1.9%	Prevalence (n/N)	13.3 (13/98)	11.2 (10/89)	44 (12.9)	6.8%	Prevalence (n/N)	2.3 (1/44)	9.1 (4/44)
			Titre (IQR)	25.8 (13.7–47.0)	14.37 (6.6–41.0)			Titre (IQR)	34.2 * (20.8–63.5)	31.1 (0.0–96.9)
1 to 2	126 (5.3)	3.2%	Prevalence (n/N)	9.2 (11/119)	13.8 (16/116)	16 (4.7)	0.0%	Prevalence (n/N)	0.0 (0/16)	12.5 (2/16)
			Titre (IQR)	20.5 (12.0–37.4)	19.7 (9.2–36.4)			Titre (IQR)	32.1 (17.8–53.2)	26.5 (4.4–49.8)
2 to 5	362 (15.3)	1.1%	Prevalence (n/N)	12.8 (42/328)	8.9 (30/336)	28 (8.2)	3.6%	Prevalence (n/N)	7.1 (2/28)	11.1 (3/27)
			Titre (IQR)	26.9 (12.8–54.0)	21.9 (11.1–40.7)			Titre (IQR)	58.1 * (35.2–67.4)	26.5 (−4.3–49.8)
5 to 10	340 (14.4)	4.4%	Prevalence (n/N)	22.5 (71/315)	24.8 (80/322)	46 (13.5)	4.4%	Prevalence (n/N)	15.2 (7/46)	15.2 (7/46)
			Titre (IQR)	42.0 (17.8–115.3)	33.9 (16.6–86.2)			Titre (IQR)	55.9 (34.2–94.6)	28.8 (−4.3–126.2)
10 to 15	233 (9.8)	5.6%	Prevalence (n/N)	29.5 (62/210)	45.8 (87/190)	54 (15.8)	5.6%	Prevalence (n/N)	14.8 (8/54)	24.5 (13/53)
			Titre (IQR)	57.3 (28.3–148.5)	74.9 (37.3–196.5)			Titre (IQR)	64.6 (37.3–112.3)	104.8 (17.5–208.3)
15 to 20	175 (7.4)	1.1%	Prevalence (n/N)	43.2 (70/162)	64.9 (85/131)	50 (14.7)	6.0%	Prevalence (n/N)	26.0 (13/50)	28.0 (14/50)
			Titre (IQR)	77.2 (29.7–322.9)	172.2 (52.4–263.4)			Titre (IQR)	71.3 (40.4–173.1)	110.1 (22.0–296.0)
20 to 25	140 (5.9)	1.4%	Prevalence (n/N)	61.7* (82/133)	76.2 (96/126)	25 (7.3)	4.0%	Prevalence (n/N)	32.0 (8/25)	36.0 (9/25)
			Titre (IQR)	211.1 (68.8–542.4)	201.4 (89.6–267.5)			Titre (IQR)	60.8 (34.2–152.1)	69.1 (0.0–325.7)
25 to 35	349 (14.7)	1.4%	Prevalence (n/N)	58.0 (182/314)	71.0 (237/334)	31 (9.1)	0.0%	Prevalence (n/N)	48.4 (15/31)	45.2 (14/31)
			Titre (IQR)	186.4 (55.4–503.4)	177.1 (71.2–259.1)			Titre (IQR)	91.1 (15.8–367.8)	195.9 (31.1–600.0)
35 to 50	325 (13.7)	1.2%	Prevalence (n/N)	70.8 (211/298)	79.4 (250/315)	25 (7.3)	8.0%	Prevalence (n/N)	64.0 (16/25)	56.0 (14/25)
			Titre (IQR)	345.9 (103.7–644.2)	183.1 (102.9–266.8)			Titre (IQR)	298.8 (76.3–1587.3)	274.7 (171.7–669.0)
>50	213 (9.0)	4.2%	Prevalence (n/N)	77.0 (147/191)	82.5 (160/194)	23 (6.7)	4.4%	Prevalence (n/N)	65.2 (15/23)	56.5 (13/23)
			Titre (IQR)	432.5 (140.3–700.9)	180.4 (111.5–264.7)			Titre (IQR)	189.4 (90.0–739.7)	348.8 (115.4–901.6)
Total	2,368	2.5%				342	4.7%			

The median titre and interquartile range (IQR) were presented.

* denotes a significant difference in the median values tested by ranksum analysis.

### IPTi study

A total of 3862 subjects were enrolled in the main study with similar numbers recruited between Korogwe and Same district sites: subject numbers at individual health facilities ranged from 421 to 499. Two thirds (66.0%) of samples were from those attending the health facility as patients, the majority of these as outpatients, and the remainder were accompanying guardians and/or siblings. Participant age ranged from infancy (4 weeks old) to 96 years. Although there were no age or gender restrictions on participation in the study, the age groups showing the greatest participation were <4 and >22 years, with an overall mean age of 24 years. Sixty six percent of participants were female, presumably due to attendance of women at antenatal clinics or as an accompanying guardian for a child. RDT parasite positivity rate was higher in the Korogwe health facilities than in Same (Table 2).

**Table 2:** Distribution of study participants by age group, sex, RDT and sero-positivity to both MSP-1_19_ and AMA-1 for health facilities in Korogwe and Same districts.

age group	total (%)	Korogwe				Total	Same			
		% female	% RDT positive	% MSP-1_19_ seropositive	% AMA-1 seropositive		% female	% RDT positive	% MSP-1_19_ seropositive	% AMA-1 seropositive
<1	348 (17.6)	50.3	4.7	9.9	18.0	240 (12.7)	50.0	0.0	2.1	9.2
1 to 2	90 (4.5)	44.4	21.1	16.9	16.9	94 (4.9)	48.9	1.1	1.1	3.2
2 to 5	161 (8.2)	54.7	24.8	20.0	35.6	135 (7.15)	61.5	0.8	1.5	2.2
5 to 10	143 (7.2)	53.2	32.4	23.1	53.9	122 (6.5)	49.2	2.5	3.4	7.4
10 to 15	94 (4.8)	56.4	17.2	36.2	79.8	126 (6.7)	64.3	0.8	3.3	22.4
15 to 20	127 (6.4)	74.8	11.1	50.0	77.8	182 (9.6)	64.8	4.1	14.4	44.8
20 to 25	172 (8.7)	83.7	7.0	54.1	84.9	160 (8.5)	79.4	0.0	23.1	48.8
25 to 35	371 (18.8)	81.1	4.6	53.4	87.3	290 (15.4)	78.9	0.4	25.6	52.1
35 to 50	273 (13.8)	72.2	2.9	57.4	86.8	283 (15.0)	77.0	1.1	36.9	66.3
>50	195 (9.9)	61.0	2.6	73.2	87.1	256 (13.6)	63.3	0.0	35.6	57.9
Total	1,974	65.3	9.8	40.7	64.1	1,888	65.9	0.9	18.6	37.7

**Figure 3:**
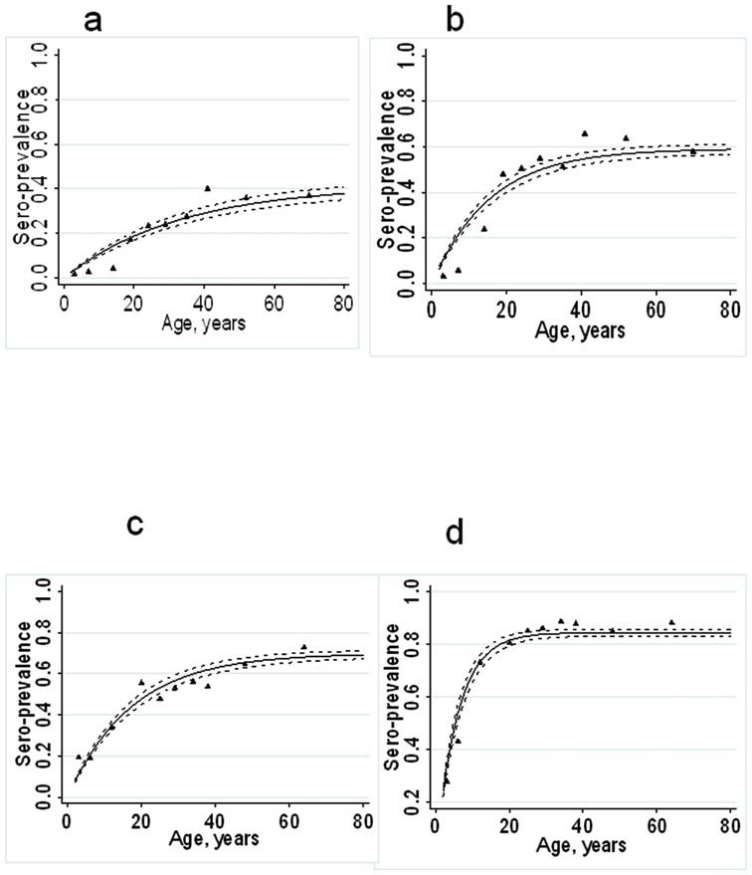
Age sero-prevalence plots for MSP-1_19_ and AMA-1 fitted by maximum likelihood with a single force of infection for the dispensaries in the Kili-IPTi study. Plot a) MSP-1_19_ Same district; b) AMA-1 Same district; c) MSP-1_19_ Korogwe district and d) AMA-1 Korogwe district. Black triangles represent observed data and black lines predicted values. Dotted black lines represent upper and lower 95% CI for the predicted SCR.

**Figure 4:**
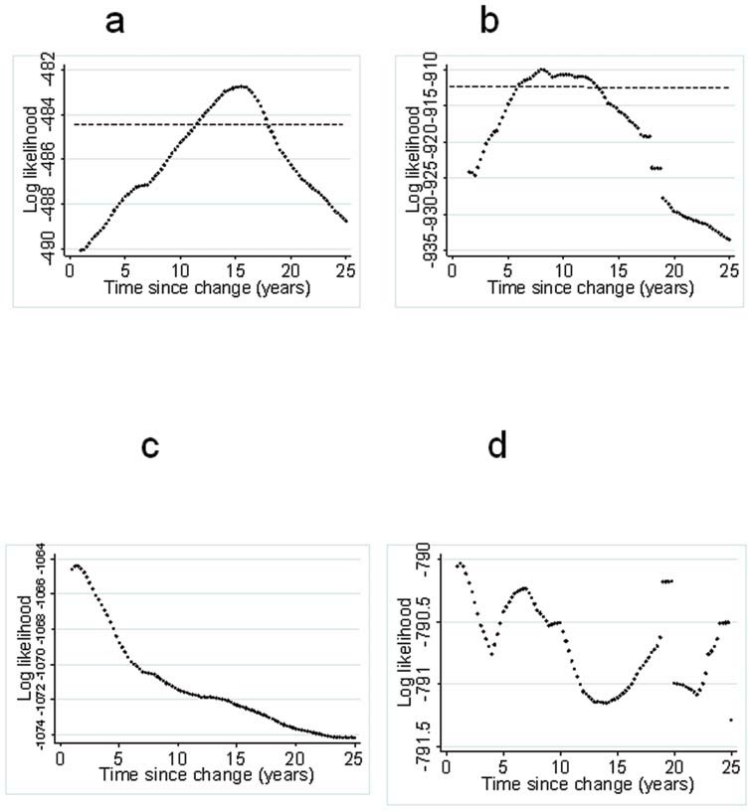
Univariate profile likelihood to evaluate the time at which sero-conversion rates changed. Same region fits are represented in a) for MSP-1_19_ fits and b) for AMA-1. The broken black line is the 95^th^ percentile of the Chi-squared on 1 degree of freedom below the maximum. The two points at which this line crosses the log-likelihood profile are used to determine an approximate 95% confidence interval for the time since the change in SCR i.e. 11–18 years for MSP-1_19_ and 6 to 14 years for AMA-1. The equivalent plots for Korogwe are shown in c) for MSP-1_19_ and d) for AMA-1.

**Figure 5:**
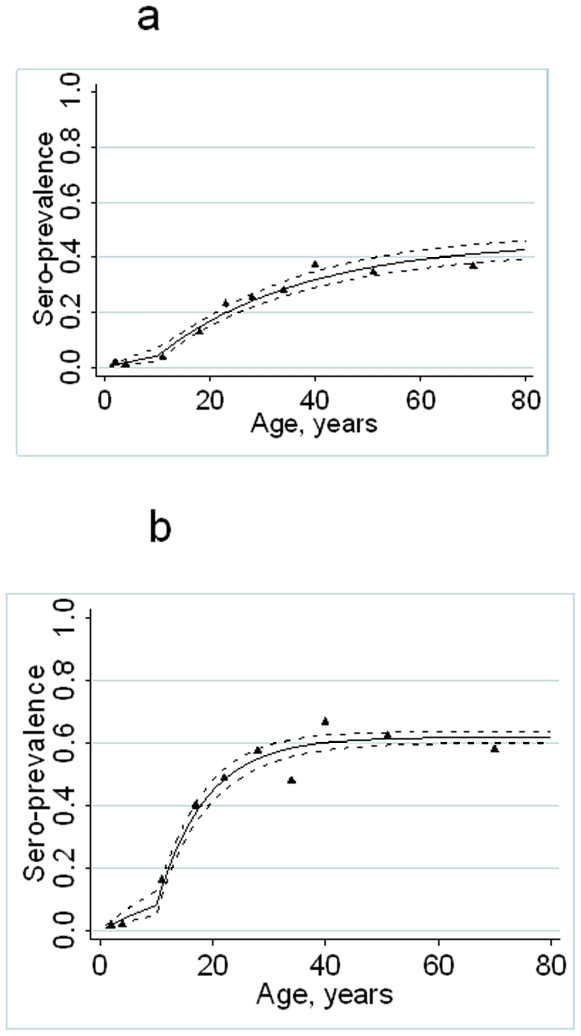
Age sero-prevalence plots for MSP-1_19_(a) and AMA-1 (b) fitted by maximum likelihood with a two forces of infection for Same district. Black triangles represent observed data and black lines predicted values. Dotted black lines represent upper and lower 95% CI for the predicted SCR.

ELISA was performed on 3859 filter paper samples for MSP-1_19_ and 3862 samples for AMA-1. Sero-positivity to both antigens increased with age and was higher in the Korogwe health facilities (MSP-1_19_ and AMA-1 both p<0.001; Table 2). Antibody positivity rates were not significantly different between sick children and children who were well (MSP-1_19_ 12.0% vs 8.6% p = 0.1, AMA-1 17.2 vs 19.3 p = 0.8). Age sero-prevalence plots for the Same and Korogwe regions are shown in Figure 3. Visual assessment of the plots for the Same dispensaries indicated a poor fit of the model, for younger age groups, where sero-prevalence was lower than predicted. When a model, which allowed for a single change in sero-conversion rate was fitted to the combined data from the 4 Same health facilities, the best fitting model was that with the change between the two SCRs occurring approximately 15 years previously (CI 11–18) (i.e.1992) according to the MSP-1_19_ data (Figure 4a) and 8 years previously (CI 6–14) (i.e. 1999) according to the AMA-1 data (Figure 4b). We chose a model compatible with both antigens which assumed that the SCR in Same changed 10 years previously, which had a significantly better fit than the model that assumed the SCR had remained constant (likelihood ratio test for MSP-1_19_ Χ^2^ = 9.6 p = 0.002 and AMA-1 Χ^2^ = 45.4 p<0.0001). Sero-prevalence plots for Same assuming a change in SCR 10 years previously are shown in Figures 5a and 5b, for MSP-1_19_ and AMA respectively. A change in SCR was observed for all 4 health facilities in Same (Table 3) but was not observed for any of those in Korogwe region (Figures 4c & 4d)(Similar analysis was conducted on pilot survey data which showed the best fitting model with a single conversion rate).

**Table 3:** Sero-conversion rates and EIR equivalents for MSP-1_19_ and AMA-1 by district and by individual health facility.

Site		MSP				AMA			
		Sero conversion rate	CI	EIR equivalent	CI	Sero conversion rate	CI	EIR equivalent	CI
Same (all)	previous	0.025	0.021–0.03	0.6	0.4–0.9	0.066	0.057–0.078	0.6	0.4–1
	Current	0.005	0.002–0.01	0	0–0.1	0.01	0.006–0.016	0	0–0
Same DH	previous	0.02	0.015–0.028	0.4	0.2–0.8	0.043	0.031–0.06	0.2	0.1–0.5
	Current	0.002	0–0.013	0	0–0.2	0.011	0.004–0.027	0	0–0.04
Kisiwani	previous	0.044	0.031–0.062	2	1–4.3	0.133	0.097–0.182	4.7	1.9–11.7
	Current	0.003	0–0.022	0	0–0.5	0.005	0.001–0.018	0	0–0.01
Gonja Maore	previous	0.023	0.009–0.06	0.5	0.1–4.1	0.071	0.049–0.103	0.8	0.3–2.2
	Current	0.008	0.003–0.02	0.06	0–0.4	0.008	0.003–0.023	0	0–0.03
Ndungu	previous	0.032	0.024–0.044	1.1	0.6–2.1	0.071	0.049–0.101	0.7	0.26–2.1
	Current	0.003	0–0.013	0	0–0.3	0.014	0.007–0.031	0	0.001–0.07
Korogwe (all)		0.04	0.037–0.044	1.7	1.4–2	0.126	0.115–0.138	4	3–5.3
Magunga		0.03	0.025–0.035	0.9	0.6–1.3	0.075	0.064–0.089	0.9	0.5–1.5
Majengo		0.034	0.029–0.041	1.2	0.8–1.7	0.092	0.077–0.11	1.6	0.9–2.7
Mnyuzi		0.063	0.053–0.074	4.4	3.1–6.2	0.188	0.155–0.228	12.8	7.2–22.5
Magasin		0.041	0.034–0.048	1.8	1.2–2.5	0.209	0.171–0.255	17.4	9.8–31.1

**Figure 6:**
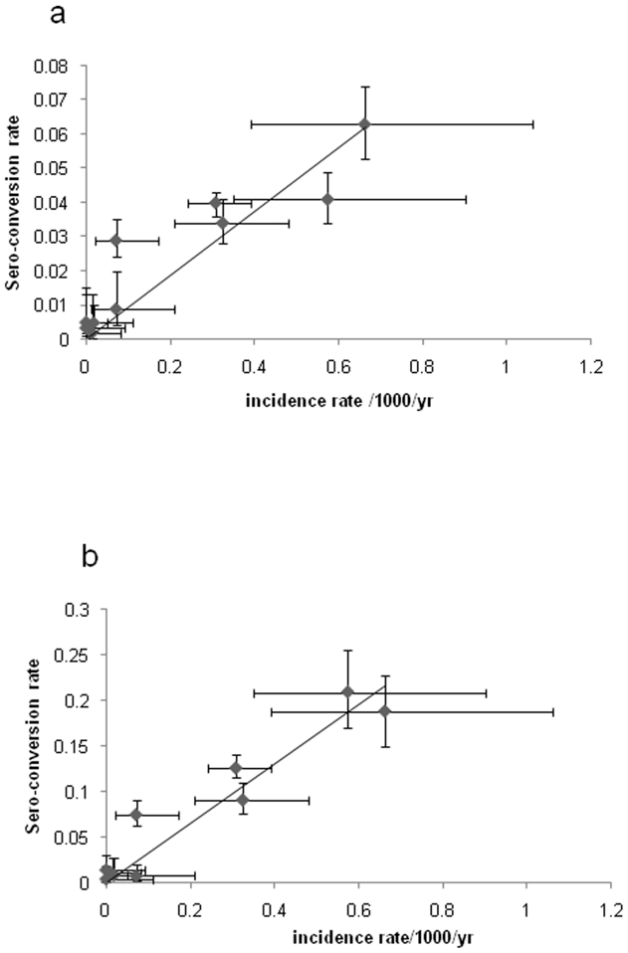
Current sero-conversion rates and clinical malaria incidence rates in the IPTi placebo cohort for each health facility: (a) for MSP-1_19_ and (b) for AMA-1. Vertical bars indicate the 95% CI for SCR and horizontal bars indicate the 95% CI for malaria incidence. Fitted lines represent linear regression plots. R^2^ values for MSP-1_19_ and AMA are 0.78 and 0.91, respectively.

The estimated SCR for each health facility, including both the current estimate and the historical estimates for those in Same, for each antigen, along with the respective estimates of EIR, are shown in Table 3. SCRs for the different antigens were correlated (Pearson correlation coefficient r = 0.88, CI 0.78–0.97). Excluding from the analysis any individuals who were positive for malaria infection by RDT did not affect the SCR estimate. In Korogwe, the SCRs calculated using all samples were 0.04 (CI 0.037–0.044) for MSP-1_19_ and 0.126 (CI 0.115–0.138) for AMA-1 (Table 3). The corresponding values when RDT positive samples were excluded from the analysis were 0.037 (0.033–0.041) for MSP-1_19_ and 0.114 (0.103–0.126) for AMA-1. SCRs were not significantly different when calculated for patients or accompanying individuals (Table 4). Our estimates of SCR demonstrate that there is considerable heterogeneity in transmission across the study site and suggest that the current upper estimates of transmission (i.e. the upper 95% confidence limit) in the study area range from less than 0.1 to more than 30 infectious bites per person per year. Estimated SCR and EIR equivalents show a trend for transmission to be higher in communities closer to the Indian ocean, as we have documented previously [27]. In the Same dispensaries our SCR estimates indicate a 2-fold (Same District Hospital) to ∼20-fold (Kisiwani) decrease in transmission in recent years. Finally, for each health facility, current SCR values were highly correlated with clinical malaria incidence rates among infants in the placebo (untreated) cohort of the contemporaneous IPTi study conducted at the same sites [26] (Pearson correlation coefficient MSP-1_19_ r = 0.78, p<0.01; AMA-1 r = 0.91, p<0.001) (Figure 6a & 6b).

**Table 4:** Sero-conversion rates for MSP-1_19_ and AMA-1 by district and by individuals attending the health facility as health seekers or as companions.

Site	Antigen		Service seekers	Companions
Same	MSP-1	previous	0.024 (0.018–0.032)	0.028 (0.023–0.034)
		current	0.005 (0.002–0.012)	0.000 (0.000–0.000)
	AMA-1	previous	0.066 (0.052–0.082)	0.068 (0.053–0.087)
		current	0.011 (0.006–0.018)	0.008 (0.003–0.025)
Korogwe	MSP-1		0.043 (0.039–0.049)	0.034 (0.030–0.040)
	AMA-1		0.134 (0.119–0.152)	0.116 (0.099–0.136)

## Discussion

In this study we have used the prevalence of antimalarial antibodies in samples collected from health centre attendees to generate measures of MTI. Age-specific sero-prevalence rates were used to estimate sero-conversion rates (SCR), which have previously been shown to correlate closely with the gold standard measure of malaria transmission, EIR [23]. Consistent with previous studies in the same area [27], [31], estimated transmission intensity varied from village to village according to altitude and proximity to the Indian Ocean and varied more than 10-fold between geographically adjacent communities (Korogwe EIR equivalent range between 1 and 14 ib/p/yr). Importantly, the data point to a recent marked reduction in malaria transmission in one of the study sites; our best estimate is that EIR in Same region has fallen by approximately 90% in the last 10 years, in line with estimates generated from conventional malariometric parameters from nearby Kenya [5], [8], [32]. Interpretation of the relationship between SCR and EIR needs to allow for variation in the estimates for both measures. Similarly, caution will be required when comparing these SCR estimates between different areas and health systems until the degree of bias in an estimate is more fully understood. However, the measures of MTI generated by this relatively quick and simple approach could find widespread application as an operational tool for malaria control programmes.

There are a number of potential limitations associated with recruitment of individuals for sero-prevalence studies via health centres. Firstly, attendance for childhood immunisation or antenatal clinics means that (as seen here) the cohort is likely to be biased towards recruitment of children under the age of 5 years and women of child bearing age. Over-representation of small children and their mothers was observed in the pilot study and may have contributed to the observed differences in anti-MSP-1_19_ seroprevalence and titres between the health facility and community surveys. To compensate for this, in the main study, field workers were encouraged to sample accompanying family members as well as those seeking health care and this allowed sufficient numbers of individuals aged from 5 to 20 years to be recruited at each site to allow estimates of SCR to be obtained with a sufficient degree of precision. In future studies, numbers in this age group could also be augmented from surveys in local schools [33]. Secondly, and perhaps more importantly, many health centre attendees are ill and, depending on the transmission intensity, a substantial proportion of these will have active malaria infections which could, in theory, influence sero-prevalence rates. In the pilot study there were, predictably, more parasite positive individuals recruited at the health centres than in the cross sectional surveys and in both the pilot study and the main study parasitaemic individuals were 2–3 times more likely to be antibody positive than aparasitaemic individuals. In the main study, however, excluding these RDT positive individuals from the analysis resulted in only marginally lower SCR suggesting that concurrent patent malaria infections do not bias SCR estimates. One reason for this may be that sero-prevalence may also be affected by sub-patent parasite carriage (i.e. parasitaemia that is below the limit of detection by microscopy and RDT's). Sub-patent infections are far more frequent than patent infections, even at quite low transmission intensities [29], [34]–[36], and it is probable that similar levels of asymptomatic carriage are present in health facility attendees and in participants in cross-sectional surveys. Further work is underway to investigate the impact of patent and subpatent parasitaemia on SCR. Future studies will also need to evaluate the impact of HIV infection on malaria seroprevalence. There are few data relating to the effect of HIV infection on anti-malarial antibody levels and these data are inconclusive.HIV infection may reduce anti-malarial antibody concentrations in pregnant women but has no effect malaria specific antibody responses in HIV infected neonates [37], [38]. Finally, although most health facilities have defined catchment areas, with the majority of attendees coming from within this area [39], SCR estimates for some facilities may be skewed by recruitment of individuals who come from further away. This is most likely to be the case for referral facilities and for particularly well-resourced health facilities on major transit routes. Ideally, such facilities would not be selected for survey; if this is unavoidable then a simple question on village of residence could be used to refine the catchment area or to filter out attendees from outside the local community.

Whilst a health facility survey has less epidemiological rigour than a full cluster sampled household based survey, it does have several advantages. These surveys are rapid: SCR and MTI data were available from the 8 sites within 8 weeks of starting the surveys. The operational requirements were minimal: training and routine monitoring of health centre staff was performed by 2 project staff travelling between facilities by motorbike and ELISA assays were all performed by one laboratory technician. This operational ease is reflected in the cost; a crude comparison of costs between the health centre surveys (conducted in 2007) and cross sectional surveys conducted in the same area (in 2001) [27] suggests a 5- to 10- fold cost saving (US$1–2 per person compared with US$10–15 per person). These operational benefits are in addition to the benefits of the increased sensitivity and relative stability of antibody responses over time compared with using parasite rate or EIR to determine transmission intensity and the fact that surveys can be carried out at any time of year rather than being, restricted to ‘high’ or ‘low’ transmission seasons [24]. With malaria control activities needing to become increasingly focused there is a clear case for making more use of routine health facility data to provide local relevant estimates of transmission [40]. Training and equipment to carry out health facility serological surveys could be built into the strengthening of the health centre system, providing important information on the population at risk from malaria and guiding more detailed surveys to identify high prevalence communities that may require greater resources from malaria control programmes and low prevalence communities that need to be monitored for re-emergence of transmission.

A very important benefit of the sero-prevalence approach to estimating malaria transmission is the ability to detect historical changes in MTI. Whilst the step-change in SCR in the four health facilities in Same district is highly indicative of a marked change in transmission over the last decade; in theory the same pattern could be seen if there was an age-related change in risk of malaria exposure – for example by virtue of travel to or employment in an area of differing transmission [36]. Whilst, from our knowledge of the socio-economic and demographic characteristics of this community, this seems unlikely we need to formally exclude this possibility. Fortunately, two of the health centres studied here serve villages for which we have SCR estimates from our previous village-based cross-sectional surveys in 2001 [23]. In the village of Kadondo, which is in the catchment of Gonja Maore health facility in Same district, the AMA-1 SCR from 2001, was 0.112 (CI 0.09–0.13) which is similar to our current estimate of SCR prior to 1998 (SCR 0.071, CI 0.049–0.103) and noticeably higher than our current estimate for post-1998 (SCR 0.008, CI 0.003–0.023). Moreover, there was no evidence of a step-change in SCR in 10–15 year olds in the data collected in 2001, arguing against age-related behavioural changes as an explanation for changing SCR. Taken together with both regional [32] evidence of a decrease in malaria transmission and a 70% decline in slide positivity in outpatients to Same hospital between 2002 and 2006 [41], these observations buttress our conclusion of a drop in transmission intensity in Same District in the mid- to late-1990s.

In Korogwe district, the 2001 SCR value for AMA-1 for Mgila, which lies within the catchment of Magasin health facility, was 0.187 (CI 0.16–0.22) which compares well with the current estimate of 0.21 and suggests that malaria transmission may not have changed here so significantly over the last few years. In contrast, although no step-change in SCR was evident, the paired MSP-1_19_ estimates for Mgila in 2001 and Magasin in 2008 show a decrease in SCR from 0.13 (0.9–1.6) to 0.04 (0.03–0.05). Given the proximity of Same and Korogwe districts, and mounting regional evidence for change in transmission, it seems likely that transmission is also gradually declining in Korogwe district. The differences between the AMA-1 and MSP-1_19_ estimates in detecting changes in transmission may be linked to differences in seroconversion and reversion rates and this could also explain the differences observed in the pilot study. It is likely that seroconversion and reversion rates are different for different antigens, possibly reflecting their inherent immunogenicity, subclass dependent half-life, polymorphism etc. AMA-1 appears to be more immunogenic than MSP-1_19_, and anti-AMA-1 titers tend to be higher than those for MSP-1_19_, suggesting that seroconversion may be faster and sero-reversion may be slower for AMA-1 than for MSP-1_19_. However, the extent to which these differences influences estimates of malaria transmission is not yet clear and is the subject of ongoing analysis in our group.

The reasons for the decline in MTI are unclear. The Same area was once hyperendemic for malaria and was part of the Pare-Taveta malaria control scheme of the 1950s [42]; although this scheme was short-lived it was highly effective and, transmission was still lower 20 years on [43]. In recent years we have found no evidence of concerted, systematic interventions in the area. Bed net use was 40% in 2001 with 10% of these insecticide treated [27]. Whilst ITN use has undoubtedly increased since then [44] it is unlikely to explain this major drop in transmission. Sociological factors, such as improved health services [39], increasing access to health interventions and better housing, as well as meteorological parameters (e.g. mean annual rainfall has dropped from 600 mm pa to 500 mm pa in the last 15 years) are all likely to have an effect on transmission.

In summary, we have used a rapid, health centre-based, serological survey to provide local estimates of malaria transmission intensity. These estimates are consistent with cross-sectional data collected previously in the same communities, emphasise the heterogeneity in transmission within a relatively restricted geographical area and have allowed quantification of the recent drop in malaria transmission in the region. The envisaged value of this serological approach [11], [12], [45] is being born out and the fact that these data can be generated quickly, cheaply and easily through the existing health infrastructure represents a potentially important innovation for implementation and monitoring malaria control activities.
